# Antioxidants inhibit low density lipoprotein oxidation less at lysosomal pH: A possible explanation as to why the clinical trials of antioxidants might have failed

**DOI:** 10.1016/j.chemphyslip.2018.03.001

**Published:** 2018-07

**Authors:** Feroz Ahmad, David S. Leake

**Affiliations:** Institute of Cardiovascular and Metabolic Research, University of Reading, Reading, United Kingdom

**Keywords:** Low density lipoproteins, Macrophages, Lipid peroxidation, Probucol, Cysteamine, *N,N*'-diphenyl-1,4-phenylenediamine

## Abstract

Oxidised low density lipoprotein (LDL) was considered to be important in the pathogenesis of atherosclerosis, but the large clinical trials of antioxidants, including the first one using probucol (the PQRST Trial), failed to show benefit and have cast doubt on the importance of oxidised LDL. We have shown previously that LDL oxidation can be catalysed by iron in the lysosomes of macrophages. The aim of this study was therefore to investigate the effectiveness of antioxidants in preventing LDL oxidation at lysosomal pH and also establish the possible mechanism of oxidation. Probucol did not effectively inhibit the oxidation of LDL at lysosomal pH, as measured by conjugated dienes or oxidised cholesteryl esters or tryptophan residues in isolated LDL or by ceroid formation in the lysosomes of macrophage-like cells, in marked contrast to its highly effective inhibition of LDL oxidation at pH 7.4. LDL oxidation at lysosomal pH was inhibited very effectively for long periods by *N,N*'-diphenyl-1,4-phenylenediamine, which is more hydrophobic than probucol and has been shown by others to inhibit atherosclerosis in rabbits, and by cysteamine, which is a hydrophilic antioxidant that accumulates in lysosomes. Iron-induced LDL oxidation might be due to the formation of the superoxide radical, which protonates at lysosomal pH to form the much more reactive, hydrophobic hydroperoxyl radical, which can enter LDL and reach its core. Probucol resides mainly in the surface monolayer of LDL and would not effectively scavenge hydroperoxyl radicals in the core of LDL. This might explain why probucol failed to protect against atherosclerosis in various clinical trials. The oxidised LDL hypothesis of atherosclerosis now needs to be re-evaluated using different and more effective antioxidants that protect against the lysosomal oxidation of LDL.

## Highlights

• The initial oxidation of LDL by iron under lysosomal conditions occurs in the hydrophobic core where probucol has limited access.
• At lysosomal pH hydroperoxyl radical (HO_2_•) would be the main oxidising species which can be scavenged by cysteamine.
• Our findings here might explain why probucol failed to protect against atherosclerosis in the PQRST Trial.
• Lysosomotropic antioxidants might be the effective antioxidants to test the oxidised LDL hypothesis of atherosclerosis.

## 1 Introduction

Oxidation of low density lipoprotein (LDL) was widely believed to be a critical step in the pathogenesis of atherosclerosis, the underlying cause of myocardial infarctions and thrombotic strokes (Steinberg and Witztum, 2010). The oxidised LDL hypothesis proposes that cells in the arterial wall oxidise LDL in the extracellular space and then take it up rapidly, leading to the formation of the foam cells characteristic of atherosclerosis (Steinberg and Witztum, 2010). Two key problems with this conventional view are that the oxidation is inhibited by low concentrations of interstitial fluid (or serum) (Dabbagh and Frei, 1995), and that large clinical trials have shown no protection by antioxidants against cardiovascular disease (Steinberg and Witztum, 2002). To address the shortcomings in the conventional LDL oxidation theory, we proposed that nonoxidatively modified LDL is rapidly endocytosed by macrophages and then oxidised within lysosomes (Wen and Leake, 2007). We showed that 7 days after taking up mechanically-aggregated (vortexed) LDL, mouse J774 cells (a macrophage-like cell line) and human monocyte-derived macrophages (HMDM) generated ceroid (an advanced lipid oxidation product present in human atherosclerotic lesions) in their lysosomes (Wen and Leake, 2007). Furthermore, there was an increased production of oxysterols (e.g. 7-ketocholesterol) in the J774 cells when incubated with nonoxidized acetylated LDL which is rapidly endocytosed by macrophages, and then incubated in the absence of extracellular lipoproteins. Chloroquine, a weak base that concentrates in lysosomes and increases their pH, inhibited the oxidation of LDL, suggesting that the oxidation takes place at acidic pH. It was further shown that the oxidation in lysosomes is mediated by iron which is highly effective in oxidising LDL at pH 4.5, the approximate pH of lysosomes, but very poor at doing so at pH 7.4 in a simple buffer (Satchell and Leake, 2012; Wen and Leake, 2007).

Iron is a transition metal capable of redox cycling between the oxidation states of Fe^2+^ and Fe^3+^. Whether iron in lysosomes exists in ferric or ferrous state has been a matter of discussion, (Collins et al., 1991) and both might be present (Meguro et al., 2005), but because of the reducing environment within lysosomes the ferrous oxidation state of iron is likely to be favoured (Terman and Kurz, 2013). Various theories have been proposed for the mechanism of LDL oxidation by iron. Some suggest that iron may be able to catalyse the oxidation of LDL by the production of hydroxyl radicals from hydrogen peroxide (Halliwell and Gutteridge, 1986), while others believe that superoxide ion is required for the initiation of LDL oxidation (Lynch and Frei, 1993). It is also believed that iron may be able to initiate the oxidation of LDL by reacting with pre-existing lipid hydroperoxides (LOOH) in LDL particles (Esterbauer et al., 1990).

These mechanisms are based on reactions occurring at physiological pH (Lynch and Frei, 1993), at which iron is practically insoluble and precipitates unless bound to various molecules, and might not be indicative of the reactions that occur at lysosomal pH. We show here that antioxidants that have been used to test the oxidised LDL hypothesis do not inhibit the oxidation of LDL effectively at lysosomal pH and hence might not be a true test for this hypothesis. We propose another antioxidant, cysteamine, which accumulates in lysosomes and inhibits the oxidation of LDL effectively at acidic pH, which might provide a better test for the oxidised LDL hypothesis.

## 2 Materials and methods

Chemicals and reagents used in this study were purchased from Sigma-Aldrich, Dorset, UK, or Fisher Scientific Ltd, Loughborough, UK, unless otherwise stated. Solutions were prepared using ultrapure water generated from a Barnstead Nanopure system. Organic solvents were HPLC or molecular biology grade.

### 2.1 LDL isolation

Blood was taken from healthy volunteers after overnight fasting using EDTA (final concentration 3 mmol/L) as the anticoagulant. LDL (1.019–1.063 g/mL) was isolated from the plasma by sequential density ultracentrifugation at 4 °C, as described previously (Wilkins and Leake, 1994). LDL was stored in the presence of 100 μmol/L EDTA in the dark at 4 °C and used within 1 month.

### 2.2 Aggregation of LDL with sphingomyelinase

LDL was aggregated by incubating native LDL (2 mg protein/mL) with sphingomyelinase (Bacillus cereus, catalogue number S9396-25UN, Sigma) at 10 mU/mL (Walters and Wrenn, 2010) until the attenuance (absorbance plus light scattering) at 680 nm increased from about 0.0017–0.027. Sphingomyelinase-aggregated LDL (SMase-LDL) was dialysed against a phosphate buffer containing EDTA and sterilised by membrane filtration (0.45 μm).

### 2.3 Measurement of conjugated dienes

LDL (50 μg LDL protein/ml) was oxidised with freshly dissolved FeSO_4_ (5 μmol/L) or FeCl_3_ (5 μmol/L) at 37 °C in a NaCl/sodium acetate buffer (NaCl 150 mmol/L, sodium acetate 10 mmol/L; pH 4.5) in capped quartz cuvettes and conjugated dienes were monitored in the presence or absence of antioxidants using a method based on that of Esterbauer et al. (Esterbauer et al., 1989). The change in attenuance at 234 nm was measured at 37 °C against reference cuvettes containing all the components except LDL. Measurements were taken at one minute intervals in a Lambda-2 6-cell or a Lambda Bio 40 8-cell spectrophotometer with UV Winlab software. The attenuance at time zero was subtracted from all values, and the time taken to reach an attenuance of 0.1 was used as a reference since this corresponds to the rapid oxidation phase (Satchell and Leake, 2012).

### 2.4 Loss of LDL-tryptophan fluorescence measurement

The decrease in lipoprotein bound tryptophan fluorescence was measured on a Cary Eclipse fluorescence spectrophotometer using the time-drive method at an emission wavelength of 331 nm, with excitation set at 282 nm (Giessauf et al., 1995). The emission and excitation slits were set at 10 nm to obtain optimal fluorescence output. LDL (50 μg LDL protein/ml) was oxidised by freshly dissolved FeSO_4_ (5 μmol/L) at 37 °C in the NaCl/sodium acetate buffer, pH 4.5 in capped quartz cuvettes and the tryptophan fluorescence was measured every 10 min.

### 2.5 Monitoring of Fe^2+^ levels using bathophenanthrolinedisulfonic acid

The ferrous iron chelator bathophenanthrolinedisulfonic acid (BP) was used to measure ferrous iron concentrations. BP forms a red color complex with Fe (II) with a molar absorption coefficient of 22,140 L mol^−1^ cm^−1^ at 535 nm (Pieroni et al., 2001). LDL (50 μg protein/ml) was oxidised with freshly prepared FeSO_4_ (5 μmol/L) in the NaCl/sodium acetate buffer in polypropylene tubes in a water bath at 37 °C. Samples of 1 mL were taken at different time points into new tubes, 30 μL of BP (10 mmol/L) was added to them and absorbance was measured immediately at 535 nm with a spectrophotometer.

### 2.6 HPLC analysis

Lipids were extracted from oxidised LDL for HPLC analysis using methanol and hexane (Kritharides et al., 1993; Satchell and Leake, 2012). The upper hexane layer was collected and evaporated at ambient temperature in a SpeedVac Concentrator System (ThermoFisher), the residue was dissolved in the relevant mobile phase before injection into the HPLC. Lipid species were separated by reverse phase HPLC in a Waters C18 column (250 mm × 4.6 mm, 5 μm particle size, 5 μm guard column) with a Agilent 1100 HPLC system. Cholesteryl esters were detected at 210 nm using an acetonitrile/2-propanol/water mobile phase (44/54/2, by volume) and a flow rate of 1.2 mL/min. Cholesteryl linoleate hydroperoxide and 7-ketocholesterol were detected at 234 nm using an acetonitrile/2-propanol/water mobile phase (50/48.8/1.2, by volume) and a flow rate of 1 mL/min. The identities of the peaks were confirmed by mass spectrometry (data not shown), and the lipids were quantified using commercially available standards.

### 2.7 Measurement of lipid hydroperoxides

Lipid hydroperoxides (LOOH) were measured using a method adapted from that described by el-Saadani et al. (1989) Standard concentrations of H_2_O_2_ were made up using pure water. Samples and standards (250 μL) were added to assay tubes in triplicate and 1 mL of color reagent (0.2 mol/L potassium phosphate, 0.12 mol/L potassium iodide, 0.15 mmol/L sodium azide, polyethyleneglycol mono[*p*-(1,1′,3,3′-tetramethyl-butyl)-phenyl]ether (2 g/L), alkylbenzyldimethylammonium chloride (0.1 g/L), ammonium molybdate (10 μmol/L); pH 6.0–6.2) was added to each tube. After leaving the tubes in the dark for 1 h, the absorbance at 365 nm was measured. The levels of lipid hydroperoxides were also confirmed by HPLC method as described above (Fig. S1).

### 2.8 Cell culture

THP-1 cells were purchased from the European Collection of Cell Cultures (Salisbury, UK), and cultured in RPMI 1640 containing L-glutamine to which was added penicillin (50 IU/mL), streptomycin (50 μg/mL) and amphotericin B (0.95 μg/mL). THP-1cells were differentiated into macrophages on 18 × 18 mm glass coverslips in 6-well tissue culture plates using 25 nM phorbol 12-myristate 13-acetate (25 nmol/L) for 72 h. The cells were then washed three times with PBS and allowed to rest for 24 h before treatment with LDL. At the end of 24 h the macrophages were incubated in medium containing either SMase-LDL or native LDL at 200 μg protein/mL, or without LDL, for 24 h. The medium was washed off 3 times with warm PBS and the incubation was continued for 7 days with the above medium but containing 10% (v/v) lipoprotein-deficient fetal calf serum (LPDS), instead of fetal calf serum. LPDS was prepared by ultracentrifugation at 115 000*g* and 4 °C for 48 h at a density of 1.25 g/mL. The medium was changed every 2 days. To study effects of antioxidants, probucol (freshly dissolved in ethanol) and cysteamine (freshly dissolved in PBS), were added to the cells every day after the LDL had been washed off. To demonstrate ceroid, cells on coverslips were fixed with 4% (w/v) paraformaldehyde in PBS, treated with ethanol and xylene for 5 min each to remove ‘soluble lipids’ and stained with Oil Red O (0.5%, w/v) (Wen and Leake, 2007; Wen et al., 2014). Staining was detected using a transmitted-light microscope equipped with a digital camera (Axioskop 2, Carl Zeiss Ltd). It was quantified with ImageJ 1.46 (National Institute of Mental Health, Bethesda, Maryland, USA) by calculating the average integrated density in five randomly positioned digital images containing a total of at least 100 cells in each slide.

## 3 Statistical analysis

Data are presented as the mean ± SEM of at least 3 independent experiments. Comparisons were made using either a paired student *t* test or one-way ANOVA with a Dunnett’s post hoc test or two-way ANOVA with a Bonferroni posthoc test, as specified. Differences were considered significant at P < 0.05.

## 4 Results

### 4.1 LDL oxidation with ferrous and ferric iron

**Fig. 1:**
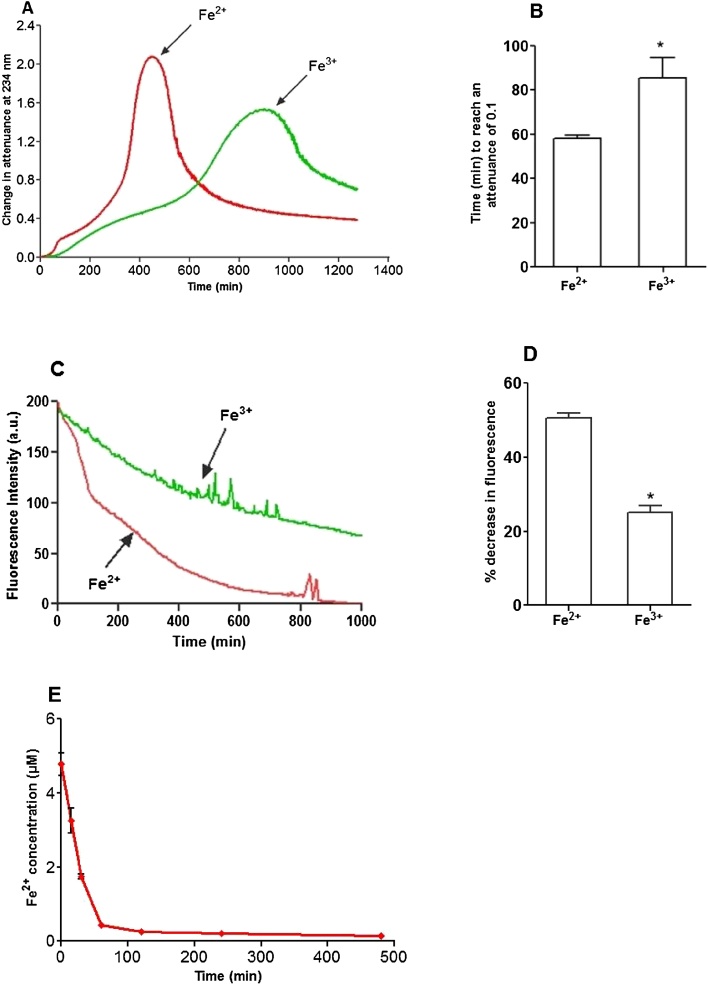
Oxidation of LDL catalysed by FeSO_4_ and FeCl_3_ at pH 4.5. LDL (50 μg protein/ml) in NaCl/sodium acetate buffer (pH 4.5) was incubated with either FeSO_4_ or FeCl_3_ (5 μmol/L) at 37 °C in quartz cuvettes. LDL oxidation was monitored by measuring the change in attenuance at 234 nm against appropriate reference cuvettes (A). Time taken to reach an attenuance increase of 0.1 is shown (B). Tryptophan fluorescence was measured every 10 min at an excitation wavelength of 282 nm and emission wavelength 331 nm in a spectrofluorometer (C). The decrease in fluorescence after 150 min is shown (D). Decrease of Fe^2+^ concentration during LDL oxidation by FeSO_4_ (E). These are representative examples of at least three independent experiments (A and C). * indicates p < 0.05; paired *t*-test (B and D).

Under lysosomal conditions most of the catalytically active iron present would be expected to be in the ferrous state (Terman and Kurz, 2013), although ferric iron has also been found to be present. (Meguro et al., 2005) The oxidation of LDL by copper at pH 7.4 is increased by the presence of pre-existing lipid hydroperoxides (Frei and Gaziano, 1993) and hence LDL which has little or no detectable pre-existing hydroperoxides should resist oxidation catalysed by iron. There were no lipid hydroperoxides detected by a tri-iodide assay or by HPLC (Fig. S1) in any of the batches of freshly isolated LDL. The same LDL was oxidised by ferrous or ferric iron, by measuring conjugated diene formation and also the loss of LDL-tryptophan fluorescence. It has been shown that tryptophan residues of apoB-100 are modified in the early stages of LDL oxidation by copper ions at pH 7.4 (Giessauf et al., 1995; Giessauf et al., 1996). Both ferrous and ferric iron were able to induce the oxidation of fresh LDL at pH 4.5, even though no pre-existing lipid hydroperoxides were detected. As expected (Satchell and Leake, 2012), LDL oxidation was significantly slower with ferric than ferrous iron. The expected lag, rapid oxidation, slow oxidation, aggregation and sedimentation phases were observed at 234 nm (Satchell and Leake, 2012) (Fig. 1A). The loss of tryptophan fluorescence was gradual with ferric iron, but there was an initial rapid phase followed by a slower phase with ferrous iron (Fig. 1C). These results suggest that iron is able to catalyse the oxidation of LDL at pH 4.5 when few if any pre-existing hydroperoxides are present. Also ferrous iron promotes LDL oxidation faster than ferric ion and therefore might be a major species involved in the oxidation of LDL in lysosomes.

### 4.2 Ferrous iron is consumed during the rapid oxidation phase

As ferrous iron might be the main iron species in lysosomes, we monitored the concentration of ferrous iron during LDL oxidation, using the ferrous iron chelator bathophenanthrolinedisulfonic acid (BP). Samples of oxidising LDL were taken at various times and added to BP and the absorbance measured immediately, to avoid any artefacts resulting from BP being present during LDL oxidation. Almost all of the ferrous iron was consumed during the first 120 min (Fig. 1E). This corresponds to the rapid LDL oxidation phase seen in the conjugated diene and LDL-tryptophan fluorescence measurements (Fig. 1A and C). This suggests that the initial rapid oxidation phase of LDL is due to ferrous ion, which is probably oxidised to the ferric state, followed by the slow oxidation phase of LDL in the presence of mainly ferric iron.

Fe^2+^ + X → Fe^3+^ + X^−^

### 4.3 Effect of hydroperoxide enrichment on LDL oxidation

**Fig. 2:**
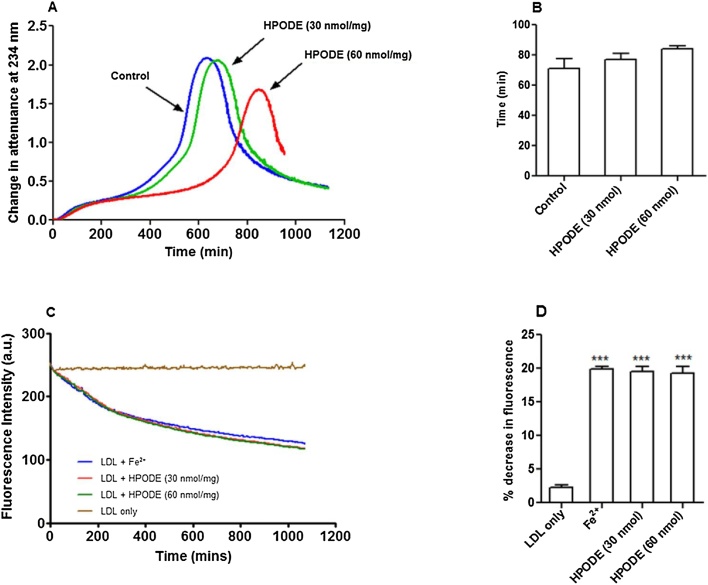
Effect of HPODE on LDL oxidation catalysed by 5 μM FeSO_4_ at pH 4.5. Native LDL and HPODE-rich native LDL (30 or 60 nmol/mg LDL protein) were incubated with 5 μM FeSO_4_ at pH 4.5 in NaCl/sodium acetate buffer (pH 4.5) at 37 °C in quartz cuvettes. Addition of lipid hydroperoxide did not increase the rate of LDL oxidation measured at 234 nm (A). There was no significant difference in the times the samples took to reach an attenuance of 0.1 (B). Addition of HPODE had no effect on the loss of LDL-tryptophan fluorescence during LDL oxidation (C). The decrease in tryptophan fluorescence at 150 min in the absence or presence of HPODE is shown (D). These are representative examples of at least three independent experiments. (*** indicates p < 0.001 compared to the control without iron).

It is believed that transition metal ions like copper and iron at pH 7.4 can initiate lipid peroxidation by breaking down pre-existing lipid hydroperoxides (Frei and Gaziano, 1993). Therefore, we wanted to confirm if the same mechanism is true under lysosomal conditions. We have shown before that adding 13(*S*)-hydroperoxyoctadeca-9Z,11E-dienoic acid (HPODE) to LDL increases the rate of LDL oxidation by copper ions at pH 7.4. (Patterson et al., 2003) As mentioned above, we used freshly isolated LDL in our experiments and checked for the presence of pre-existing lipid hydroperoxides by a tri-iodide assay for total hydroperoxides and by HPLC (Fig. S1) for cholesteryl linoleate hydroperoxide and found negligible levels. In order to study the effect of pre-existing hydroperoxides on LDL oxidation, fresh LDL was spiked with lipid hydroperoxides in the form of HPODE (30 and 60 nmol/mg LDL protein, about 4% and 8%, respectively, of the maximum content of lipid hydroperoxides in highly oxLDL) (Patterson et al., 2003) prior to oxidation with iron. Native LDL or lipid hydroperoxide-rich native LDL (50 μg protein/ml) was oxidised in NaCl/sodium acetate buffer (pH 4.5) at 37 °C with FeSO_4_. Surprisingly, addition of lipid hydroperoxide (30 nmol/mg LDL protein) to fresh LDL did not increase its oxidation with ferrous iron (Fig. 2A and B). Unexpectedly it was seen that after about 400 min oxidation slowed down in LDL spiked with 60 nmol HPODE/mg protein (Fig. 2A). HPODE also had no effect on the loss of tryptophan fluorescence with ferrous iron (Fig. 2C and D). These results indicate that pre-existing lipid hydroperoxides are not involved in initiating the iron-catalysed oxidation of LDL at pH 4.5.

### 4.4 Oxidation of LDL catalysed by iron in presence of probucol

**Fig. 3:**
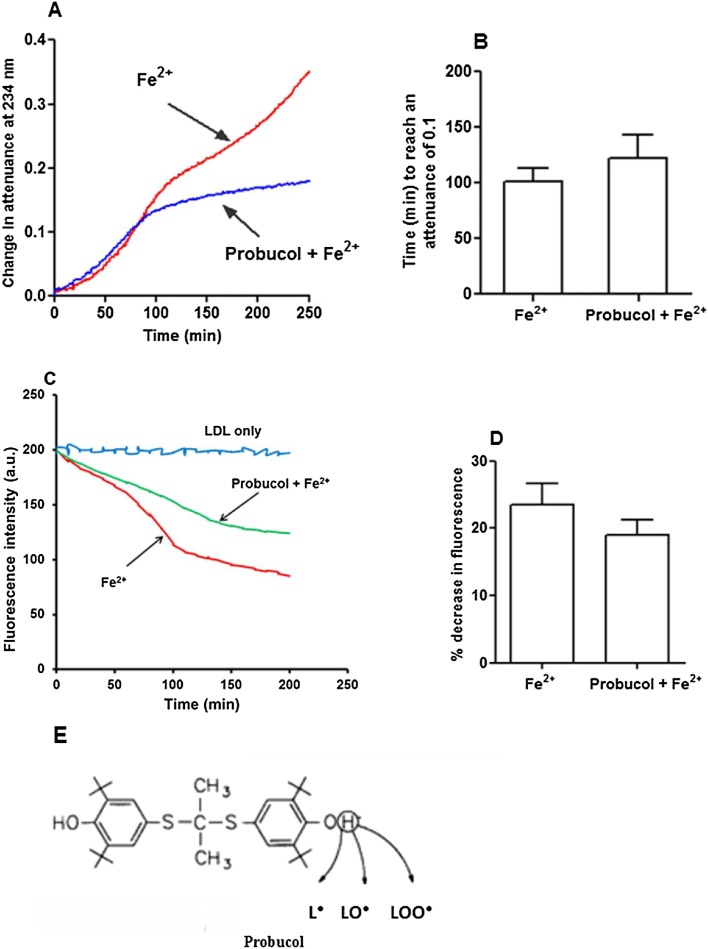
Effect of probucol on LDL oxidation catalysed by iron at pH 4.5. LDL (50 μg protein/ml) was incubated with FeSO_4_ in the presence or absence of 2 μM probucol at pH 4.5 in NaCl/sodium acetate buffer at 37 °C in quartz cuvettes; Oxidation was monitored by measuring the change in attenuance at 234 nm against appropriate reference cuvettes (A). The time required for the attenuance to reach 0.1 was not significantly increased by probucol (B). Probucol was also unable to effectively protect the initial loss of LDL-tryptophan fluorescence during incubation with iron at pH 4.5 (C). After 80 min of LDL oxidation the fluorescence intensity decrease was not significantly different (P > 0.05) from that in the presence of probucol (D). Structure of probucol (E). These are representative examples of at least three independent experiments. Mean ± SEM, paired *t*-test.

To further confirm that lipid hydroperoxides are not involved in initiating LDL oxidation, we used the chain breaking antioxidant probucol (structure shown in Fig. 3E). Probucol exerts its antioxidant activity by donating an electron or a hydrogen atom from one of its phenolic groups to a lipid radical (Jackson et al., 1991), thus inhibiting lipid peroxidation. Interestingly, probucol (2 μM) showed no initial antioxidant effect on LDL lipid oxidation induced by ferrous iron at pH 4.5 but it did slow down the oxidation after about 100 min (Fig. 3A and B). Probucol (2 μM) also did not effectively protect against the initial loss of tryptophan fluorescence during oxidation of LDL catalysed by ferrous iron at pH 4.5 (Fig. 3C and D).

### 4.5 HPLC analysis of LDL oxidation

**Fig. 4:**
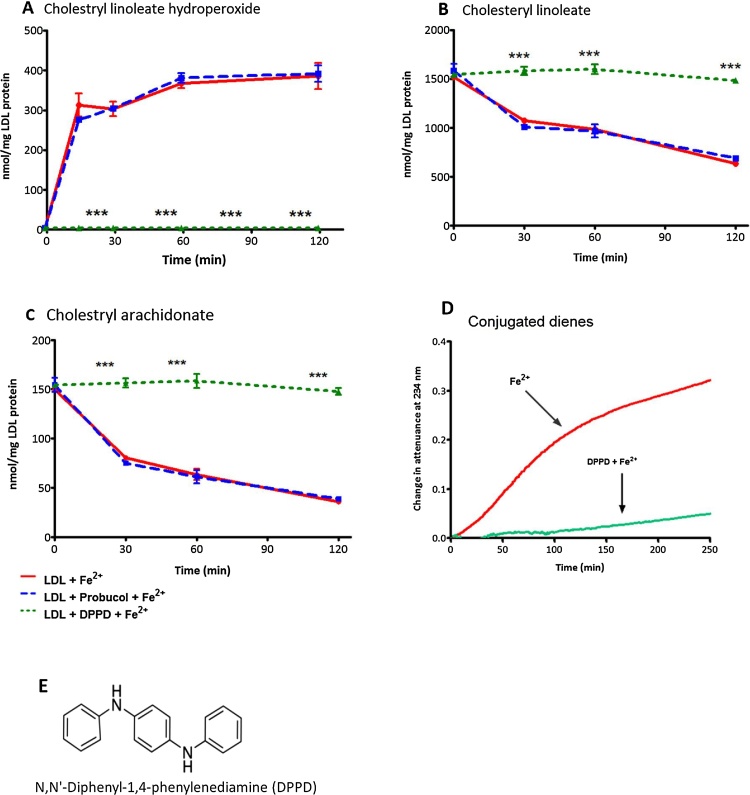
HPLC analysis of LDL oxidation catalysed by FeSO_4_ at pH 4.5. LDL (50 μg protein/mL) in NaCl/sodium acetate buffer at pH 4.5 was incubated with 5 μM FeSO_4_ at 37 °C in the presence or absence of probucol (2 μmol/L) or DPPD (2 μmol/L) and samples were taken every 30 min to be analysed by reverse-phase HPLC for cholesterol linoleate hydroperoxide (CLOOH) (A) at 234 nm and cholesteryl linoleate (B) and cholesteryl arachidonate (C) at 210 nm. Measure of conjugated diene formation in presence of DPPD (D). Structure of DPPD (E). The statistics shown are two way ANOVA followed by Bonferroni post-test (*p *< 0.0001) compared to the control. Graphs represent the mean ± the SEM of at least three independent experiments.

Probucol has been shown to concentrate in the phospholipid monolayer surrounding the hydrophobic lipid core of LDL (Bard et al., 1994) and therefore the initial oxidation of LDL seen in the presence of probucol suggests that the initial oxidation might be oxidation in the LDL core. We therefore investigated this by using reverse phase HPLC in comparison with another antioxidant *N,N*'-diphenyl-1,4-phenylenediamine (DPPD) (structure shown in Fig. 4E) which has been shown to protect against atherosclerosis in animals (Sparrow et al., 1992). DPPD is a strong antioxidant which is capable of donating an electron or a hydrogen atom from each of its secondary amine groups and prevents lipid peroxidation by scavenging LOO• (Takahashi et al., 1989)^.^ DPPD has polar surface area of 24.1 Å^2^ (PubChem) which is much lower than that of probucol which has a polar surface area of 91.1 Ǻ^2^ (PubChem) and hence DPPD would be expected to concentrate more in the hydrophobic core of LDL than does probucol. DPPD completely inhibited cholesteryl linoleate hydroperoxide (CLOOH) formation by iron at pH 4.5, whereas probucol had no inhibitory effect (Fig. 4A). DPPD completely protected against the loss of cholesteryl linoleate and cholesteryl arachidonate, whereas probucol had no effect (Fig. 4B and C) (a representative HPLC chromatogram is shown in Fig. S3). Furthermore, DPPD prevented conjugated diene formation during oxidation with iron (Fig. 4D). Probucol has limited access to the core so it is unable to prevent the oxidation of the cholesteryl esters, whereas DPPD gains access to the core of LDL and is able to completely inhibit its oxidation.

### 4.6 Lysosomal ceroid formation

**Fig. 5:**
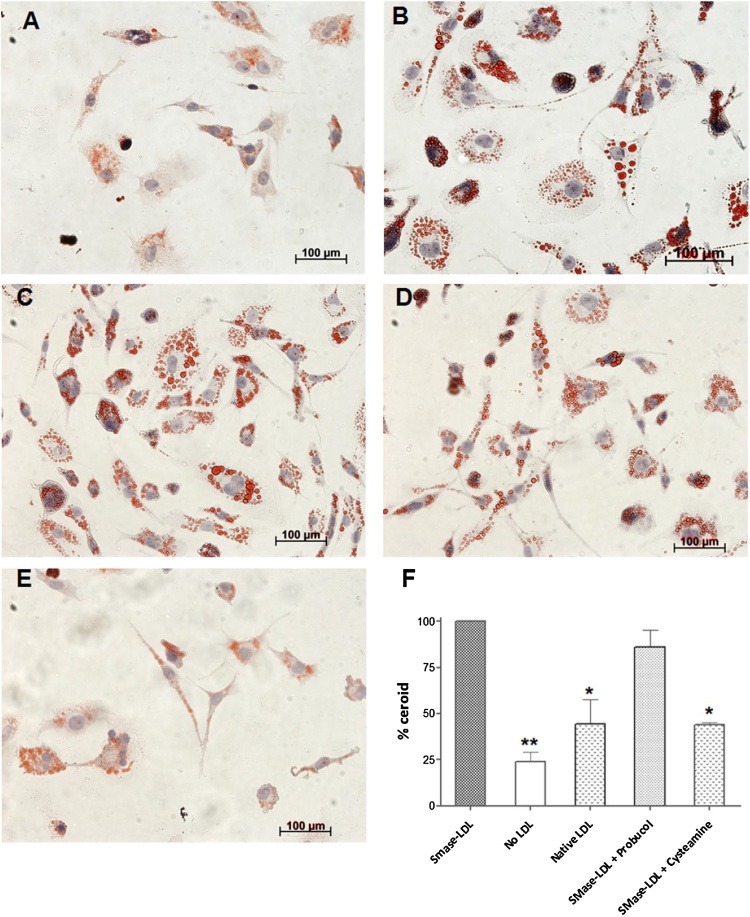
Inhibition of ceroid formation in THP-1 macrophages. THP-1 macrophage-like cells were incubated with native or SMase-LDL (200 μg protein/mL) for 24 h. They were then incubated for a further 7 days in the absence of lipoproteins in RPMI with 10% (v/v) LPDS in the presence or absence of probucol or cysteamine. A shows cells incubated without LDL or antioxidants, B shows cells incubated with native LDL only, C shows cells incubated with SMase-LDL but without antioxidants, D shows cells incubated with SMase-LDL and then probucol (10 μmol/L) and E shows cells incubated with SMase-LDL and then cysteamine (10 μmol/L). The ceroid levels in the cells were quantified using ImageJ as mean integrated density of at least 100 cells and expressed as percent decrease of cells treated with only SMase-LDL (F). Mean ± SEM of 3 independent experiments. * *p* < 0.05, ** *p* < 0.01, compared to SMase-LDL, ANOVA and Dunnett’s test.

**Fig. 6:**
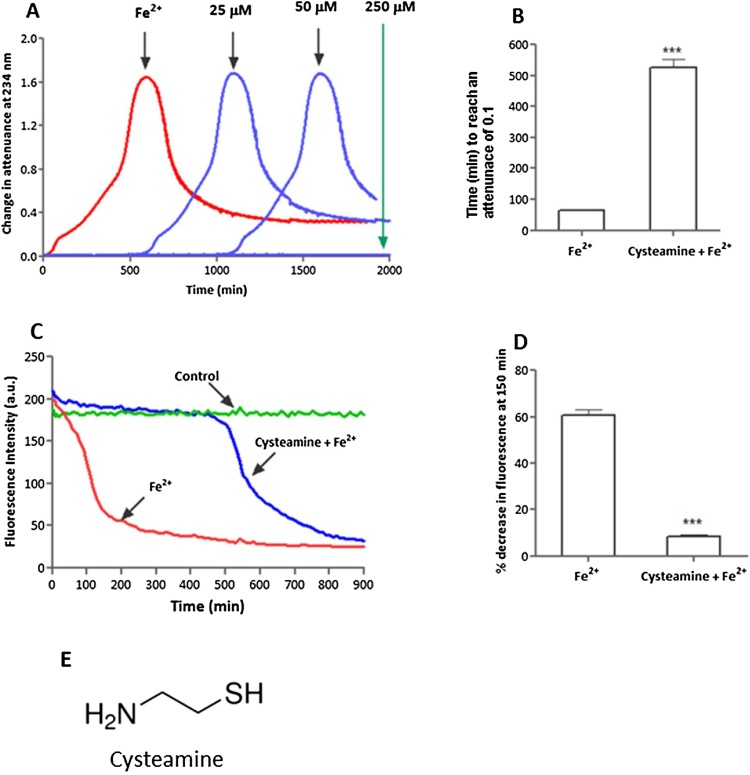
Effect of cysteamine on LDL oxidation catalysed by iron at pH 4.5. LDL (50 μg protein/ml) was oxidised in the presence or absence of cysteamine by FeSO_4_ (5 μmol/L) at pH 4.5 in NaCl/sodium acetate buffer at 37 °C. Oxidation was monitored by measuring the change in attenuance at 234 nm against appropriate reference cuvettes (A). The time required for the attenuance to reach 0.1 was significantly increased by cysteamine (25 μmol/L) (*p <* 0.001) (B) mean ± SEM, paired *t*-test, ***p < 0.001. Cysteamine (25 μmol/L) prevented the initial loss of LDL- tryptophan fluorescence on oxidation with iron (C). After 150 min of LDL oxidation with iron, the fluorescence intensity decrease was significantly less (*p *< 0.001) in the presence of cysteamine (D), mean ± SEM, paired *t*-test. (E) shows the structure of cysteamine.

To confirm the above findings of probucol’s failure as an antioxidant for the initial oxidation of LDL at lysosomal pH, we measured ceroid in the lysosomes of cultured macrophages. Ceroid is a polymerised product of lipid and protein oxidation found within foam cells in human atherosclerotic lesions (Mitchinson et al., 1985). As mentioned above, we have previously shown that mechanically aggregated LDL is rapidly taken up by human macrophages and oxidised in lysosomes generating ceroid (Wen and Leake, 2007). We have also shown that LDL aggregated by sphingomyelinase is internalised by macrophages and oxidised in lysosomes (Wen et al., 2014). Cysteamine (2-aminoethanethiol) (structure shown in Fig. 6E) is an antioxidant, which concentrates in lysosomes by several orders of magnitude, and is used in the treatment of a rare lysosomal storage disorder called cystinosis (Brodin-Sartorius et al., 2012). THP-1 macrophages were treated with native LDL or SMase-LDL (200 μg protein/ml) or without LDL for 24 h and were washed and incubated for 7 days in lipoprotein-deficient medium with or without the antioxidants, probucol (10 μmol/L) and cysteamine (10 μmol/L). The cells were then stained for ceroid after other lipids had been removed by organic solvents. Ceroid was clearly visible in the form of Oil Red O stained, irregularly shaped granules in cells treated with SMase-LDL (Fig. 5C ) (Mitchinson, 1982). Little ceroid was present in cells that had been incubated without LDL (Fig. 5A), but was increased in cells incubated with native LDL (p < 0.05) (Fig. 5B). There was no significant decrease in the ceroid development in cells incubated with probucol (Fig. 5D), but cells which were treated with cysteamine (Fig. 5E) showed a significant reduction in ceroid formation. These results show that lysosomal oxidation of LDL is not prevented by probucol but can be inhibited by the lysosomotropic drug cysteamine.

### 4.7 Effect of cysteamine on LDL oxidation catalysed by iron at lysosomal pH

**Fig. 7:**
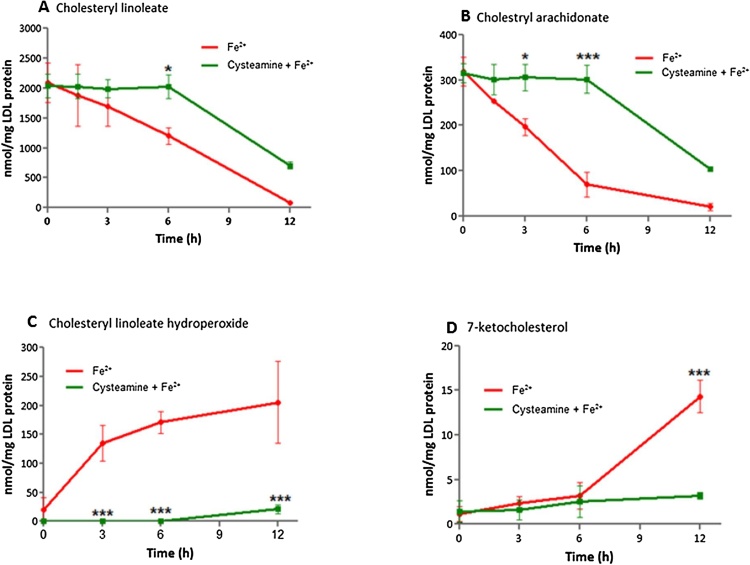
HPLC analysis of LDL oxidation catalysed by FeSO_4_ with or without cysteamine. LDL (50 μg protein/ml) was incubated with FeSO_4_ (5 μmol/L) in the presence or absence of cysteamine at pH 4.5 in NaCl/sodium acetate buffer at 37 °C. Samples were analysed by reverse-phase HPLC for cholesteryl linoleate (A), cholesterol arachidonate (B) and cholesteryl linoleate hydroperoxide (C) and 7-ketocholestrol (D). The statistics shown are two way ANOVA followed by Bonferroni post-test (*p < 0.05, ***p < 0.001) compared to the control. Graphs represent the mean ± the SEM of at least three independent experiments.

As cysteamine inhibited ceroid formation, we investigated the effect of cysteamine on LDL oxidation catalysed by iron at pH 4.5. Cysteamine showed a concentration-dependent inhibition of LDL oxidation catalysed by iron with 25 μmol/L preventing oxidation for about 8 h and 250 μmol/L for over 30 h (Fig. 6A and B). The lower concentration was chosen to conduct further experiments. Cysteamine (25 μmol/L) concentration decreased the oxidation of tryptophan residues of apoB-100 by iron for about first 8 h (Fig. 6C and D). Furthermore, cysteamine completely prevented the loss of cholesteryl arachidonate (Fig. 7A) and cholesteryl linoleate (Fig. 7B) for 6 h and also prevented the formation of cholesteryl linoleate hydroperoxide (Fig. 7C) and 7-ketocholsterol (Fig. 7D) for 6 h. These results indicate that cysteamine is able to prevent the oxidation of the hydrophobic cholesteryl ester core of LDL and of nonesterified cholesterol, which is located mainly in the surface monolayer of LDL (Lund-Katz and Phillips, 1986) as well as the oxidation of tryptophan residues in apoB-100.

## 5 Discussion

We previously showed that nonoxidatively modified LDL is endocytosed by macrophages and oxidised by iron in their lysosomes (Wen and Leake, 2007). Iron levels are increased by an order of magnitude in human and animal atherosclerotic lesions (Sullivan, 2009). Iron is highly effective in oxidising LDL at pH 4.5, the approximate pH of lysosomes, but very poor at doing so at pH 7.4 in a simple buffer (Wen and Leake, 2007). Animal studies have shown a positive correlation between iron-deposition within plaques and the severity of atherosclerosis (Lee et al., 1999; Yuan et al., 1996). Low molecular weight, probably redox-active iron has been shown to be present in lysosomes of macrophage foam cells in human atherosclerotic lesions, probably because of the degradation of ferritin, the autophagy of iron-containing organelles or the phagocytosis of erythrocytes (Kurz et al., 2008). Further to this, L-ferritin levels are increased in coronary arteries from patients with coronary artery disease, indicating that iron accumulates in atherosclerotic plaques (You et al., 2003). The epidemiology of iron status and coronary heart disease is inconsistent (Sempos, 2002). This might be because measuring iron in plasma is not a good measure of iron inside macrophages. For instance, hepcidin is increased by inflammation and decreases iron in plasma but increases it in macrophages (Sullivan, 2009).

It has been proposed that transition metals may be able to initiate the oxidation of LDL by reacting with pre-existing lipid hydroperoxides in the LDL particles (Frei and Gaziano, 1993) and ferrous iron is believed to react faster with lipid hydroperoxides than does ferric iron (Esterbauer et al., 1992).

Fe^2+^ + LOOH → Fe^3+^ + LO• + OH^−^ (fast)

Fe^3+^ + LOOH → Fe^2+^ + LOO• + H^+^ (slow)

The above reactions have been proposed on the basis of rate constants for the analogous reactions of Fe^2+^ and Fe^3+^ with H_2_O_2_ and not the actual lipid hydroperoxides (Minotti and Aust, 1992). In addition, the rate of these reactions is dependent on pH and ferric iron is insoluble at pH 7.4 and precipitates (Tachiev et al., 2000).

The current study shows that the initial addition of a lipid hydroperoxide (13-HPODE) does not increase the rate of LDL oxidation with iron at pH 4.5. In fact, addition of a higher amount of 13-HPODE delayed the later stage of oxidation of LDL, maybe because it was converting Fe^2+^ to Fe^3+^, which is less effective in oxidising LDL (Fig. 1A). It has previously been shown that the rate of oxidation of LDL by copper at pH 7.4 is increased considerably by HPODE (Patterson et al., 2003). Therefore, the present data suggest that the initial oxidation of LDL induced by iron at lysosomal pH is independent of the presence of lipid hydroperoxides.

The concentration of Fe^2+^ decreased rapidly to low levels during the first 60 min of LDL oxidation at pH 4.5 (Fig. 1E), which corresponds almost exactly to the rapid phase of LDL oxidation (Fig. 1A). Our previous work has also shown that adding higher concentrations of Fe^2+^ shortened the lag phase and increased the rates of the rapid, slow, and aggregation phases of LDL oxidation, whereas adding higher concentrations of Fe^3+^ had little effect (Satchell and Leake, 2012). The formation of conjugated dienes (Fig. 1A) and decrease in tryptophan fluorescence (Fig. 1C) both occurred faster with Fe^2+^ than with Fe^3+^, again suggesting the important role of ferrous compared to ferric iron.

Probucol efficiently inhibits LDL oxidation by cells or copper at physiological pH (Parthasarathy et al., 1986), by scavenging lipid radicals (L•, LO•, LOO•) and hence inhibiting the lipid peroxidation chain reaction. The initial oxidation of LDL catalysed by iron at pH 4.5, however, was not effectively inhibited by probucol measured by either conjugated dienes (Fig. 3A, B) or loss of tryptophan fluorescence (Fig. 3C, D). In support of this, the oxidation of the cholesteryl esters in the core of LDL was not inhibited by probucol, as measured in terms of the formation of cholesteryl linoleate hydroperoxide or loss of cholesteryl linoleate or arachidonate (Fig. 5). In contrast, cholesteryl ester oxidation was inhibited entirely by the very hydrophobic antioxidant DPPD. Probucol inhibited the oxidation of LDL after about 100 min and this might be due to an antioxidant effect on the phospholipid monolayer of LDL. This suggests that the oxidation of LDL catalysed by iron at pH 4.5 starts in the hydrophobic core (containing cholesteryl esters and triacylglycerols), where DPPD would be expected to accumulate, rather than in the surface monolayer (containing mainly phospholipids and nonesterified cholesterol) where probucol mainly resides (Bard et al., 1994),

Tryptophan loss is one of the most significant oxidative changes in oxidised proteins due to the high susceptibility of this amino acid to reactive oxygen species (Ronsein et al., 2011). ApoB-100 of LDL contains 37 tryptophan residues and these have been proposed to be responsible for the initiation of LDL oxidation by copper ions (Giessauf et al., 1995). We found that there was loss of LDL-tryptophan fluorescence during iron-catalysed oxidation at pH 4.5, which was unaffected by the addition of hydroperoxides and was not prevented by probucol. Although, DPPD greatly prevented the formation of conjugated dienes (Fig. 5D), we could not investigate the effect of DPPD on the loss of tryptophan fluorescence because DPPD absorbs at 331 nm (Linschitz et al., 1967) and quenched the fluorescence of tryptophan (Fig. S2). It was also noted that the loss of LDL-tryptophan fluorescence occurs faster when LDL is oxidised with Fe^2+^ than Fe^3+^ (Fig. 1C, D). Lynch and Frei (1993) suggested that reduction of Fe^3+^ to Fe^2+^ in the presence of an exogenous reductant such as superoxide (O_2_^•−^) is essential for iron-catalysed LDL oxidation at pH 7.4.

The concentration of Fe^2+^ falls rapidly when LDL is oxidised at pH 4.5 (Fig. 1E) and is converted presumably to Fe^3+^ generating superoxide radicals (Morgan and Lahav, 2007).

Fe^2+^ + O_2_ → Fe^3+^ + O_2_^•−^

The superoxide radical undergoes protonation at pH 4.5 to form its conjugate acid, hydroperoxyl radical, which is highly reactive (De Grey, 2002).

O_2_•− + H^+^ ↔ HO_2_• (pK_a_ 4.8)

At pH 7.4, only about 0.25% of superoxide would be present as hydroperoxyl radical, whereas at pH 4.5 about 67% of it would be present as the hydroperoxyl radical. Hydroperoxyl radical is a much more potent oxidant than superoxide anion and capable of abstracting a hydrogen atom from a polyunsaturated fatty acid (De Grey, 2002) as well as tryptophan (Dubinina et al., 2002) and oxidising LDL (Bedwell et al., 1989). Superoxide anions are negatively charged and hydrophilic and might not be able to enter the core of LDL or the hydrocarbon chain region of the surface phospholipid monolayer. Hydroperoxyl radicals, as well as being more reactive than superoxide anions, are not charged and would be able to diffuse from the aqueous phase into the core of the LDL particles and abstract hydrogen atoms from the polyunsaturated fatty acyl groups of cholesteryl esters and triacylglycerols (Bedwell et al., 1989).

LH + HO_2_• → L• + H_2_O_2_

TrpH + HO_2_•  → Trp• + H_2_O_2_

Lipid radicals and tryptophan radicals would react with oxygen to form their respective peroxyl radicals. The lipid peroxyl radicals would then lead to the lipid peroxidation chain reaction.

L• + O_2_ → LOO•

LOO• + L’ → LOOH + L’•

Trp• + O_2_ → TrpOO

Antioxidants containing thiol groups are potent nucleophiles and ready to interact with electrophilic groups of reactive oxygen species (ROS) (Güngör et al., 2011). Thiols, like cysteamine are capable of scavenging the superoxide radical in both its anionic (O_2_^•−^) and protonated forms (HO_2_•) (Cardey et al., 2007).

$$\begin{array}{ccc} NH_{2}\text{-C}H_{2}\text{-C}H_{2}\text{-SH+H}O_{2}{}^{\circ} & \to & NH_{2}\text{-C}H_{2}\text{-C}H_{2}\text{-S}{}^{\circ}+H_{2}O_{2}\\ cystea\min e & & thiyl\;\;\;radical \end{array}$$

Ceroid (lipofuscin) is a final product of lipid oxidation that consists of insoluble polymerized lipid and protein complexes and is found within foam cells in atherosclerotic lesions (Mitchinson, 1982). Cysteamine inhibited lysosomal ceroid formation in human macrophage-like cells possibly by scavenging the hydroperoxyl radical in the lysosomes, whereas probucol had little effect (Fig. 5). Cysteamine was able to completely prevent lipid peroxidation for long periods of time, as measured in terms of conjugated dienes, cholesteryl linoleate hydroperoxide and 7-ketocholesterol formation and loss of cholesteryl linoleate and arachidonate, as well as oxidation of the tryptophan residues of apoB-100 of LDL (Fig. 6, Fig. 7).

**Fig. 8:**
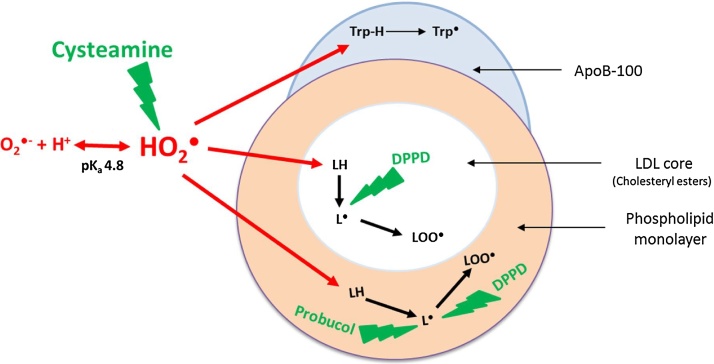
Oxidation of LDL by hydroperoxyl radicals at lysosomal pH. Iron in the lysosomes of macrophages leads to production of superoxide, which becomes protonated to the hydroperoxyl radical (HO_2_•), which attacks lipids (LH) in the both phospholipid monolayer and hydrophobic core of LDL and tryptophan residues in apoB-100. Probucol in the monolayer is unable to reach the hydrophobic core and thus cannot inhibit oxidation of cholesteryl esters, but does inhibit oxidation of the phospholipid monolayer. DPPD is more hydrophobic than probucol and inhibits oxidation in both the monolayer and core. Cysteamine scavenges hydroperoxyl or superoxide radicals in the aqueous phase and is able to inhibit the oxidation of LDL in both the core and monolayer.

Based on our data, we conclude that the initial oxidation of LDL catalysed by iron at lysosomal pH is mediated by hydroperoxyl radicals and is not dependent on pre-existing lipid hydroperoxide levels. Furthermore the oxidation of LDL in the lysosomes of macrophages is prevented by the lysosomotropic drug cysteamine, which scavenges hydroperoxyl and superoxide radicals in the aqueous phase (Fig. 8).

Probucol was initially introduced as a cholesterol-lowering drug in early 1970′s and later it was found to attenuate atherosclerotic lesion development in most animal models (Carew et al., 1987). However, the findings of The Probucol Quantitative Regression Swedish Trial (PQRST) in 1995 showed that the treatment of hypercholesterolaemic patients with probucol does not decrease the volume of femoral artery atherosclerotic lesions (Walldius et al., 1994). Two other clinical studies, The Aggressive Reduction of Inflammation Stop Events (ARISE) (Tardif et al., 2018) and Probucol Observational Study Illuminating Therapeutic Impact on Vascular Events (POSITIVE) (Yamashita et al., 2008), were conducted to assess different aspects of the treatment with probucol or its analog succinobucol, but both failed to demonstrate any significant benefits for the primary endpoints, although there were some beneficial effects. Our finding that probucol gives no protection against oxidation in the cholesteryl ester core of LDL at lysosomal pH offers an explanation about probucol’s low efficacy in the clinical trials, if LDL oxidation takes place mainly in the lysosomes of macrophages. We have previously shown that enrichment of LDL with α-tocopherol has some pro-oxidant effects in the presence of iron at lysosomal pH (Satchell and Leake, 2012). There are other possibilities to explain the low efficacy of these antioxidants in the clinical trials, however, such as low penetration into atherosclerotic lesions, an inappropriate dose or that the treatment was started too late.

DPPD inhibited LDL oxidation very effectively in both the core and monolayer. It is of interest that it protects considerably against atherosclerosis in cholesterol-fed rabbits (Sparrow et al., 1992), but it cannot be used in humans because it is mutagenic. Water-soluble antioxidants that accumulate in lysosomes, such as cysteamine, that can scavenge hydroperoxyl radicals in the acidic aqueous phase of lysosomes have potential to treat atherosclerosis in humans.

In summary, the most effective antioxidants to test the oxidised LDL hypothesis of atherosclerosis might be lysosomotropic water-soluble antioxidants or highly hydrophobic antioxidants, which can protect both the core and monolayer of LDL from oxidation, rather than moderately hydrophobic antioxidants, such as probucol, which can only protect the monolayer but not the core of LDL from oxidation.

## Conflict of interest

The authors declare no competing financial interests.

## Sources of funding

This work was supported by Felix trust and British Heart Foundation (grant no PG/15/98/31864).

## Author contributions

Both authors contributed equally to this work.
